# Voices of Women Veterans with Lower Limb Prostheses: a Qualitative Study

**DOI:** 10.1007/s11606-022-07572-8

**Published:** 2022-09-01

**Authors:** Keren Lehavot, Jessica P. Young, Rachel M. Thomas, Rhonda M. Williams, Aaron P. Turner, Daniel C. Norvell, Joseph M. Czerniecki, Anna Korpak, Alyson J. Littman

**Affiliations:** 1 Center of Innovation for Veteran-Centered and Value-Driven Care, VA Puget Sound Health Care System, US Department of Veterans Affairs, Seattle, WA USA; 2 Department of Psychiatry & Behavioral Sciences, University of Washington, Seattle, WA USA; 3 Department of Health Systems and Population Health, University of Washington, Seattle, WA USA; 4 Rehabilitation Care Service, VA Puget Sound Health Care System, US Department of Veterans Affairs, Seattle, WA USA; 5 Department of Rehabilitation Medicine, University of Washington, Seattle, WA USA; 6 Center for Limb Loss and MoBility (CLiMB), VA Puget Sound Health Care System, US Department of Veterans Affairs, Seattle, WA USA; 7 Seattle Epidemiologic Research and Information Center, VA Puget Sound Health Care System, US Department of Veterans Affairs, Seattle, WA USA; 8 Department of Epidemiology, University of Washington, Seattle, WA USA

**Keywords:** women Veterans, amputation, prostheses, qualitative research, healthcare delivery

## Abstract

**Background:**

Women Veterans with amputation are a group with unique needs whose numbers have grown over the last 5 years, accounting for nearly 3% of all Veterans with amputation in 2019. Although identified as a national priority by the Veterans Health Administration, the needs of this population have remained largely underrepresented in amputation research.

**Objective:**

To describe the experiences of women Veterans with lower extremity amputation (LEA) related to prosthetic care provision and devices.

**Design:**

National qualitative study using semi-structured individual interviews.

**Participants:**

Thirty women Veterans with LEA who had been prescribed a prosthesis at least 12 months prior.

**Approach:**

Inductive content analysis.

**Key Results:**

Four key themes emerged: (1) a sense of “feeling invisible” and lacking a connection with other women Veterans with amputation; (2) the desire for prosthetic devices that meet their biological and social needs; (3) the need for individualized assessment and a prosthetic limb prescription process that is tailored to women Veterans; the current process was often perceived as biased and either dismissive of women’s concerns or failing to adequately solicit them; and (4) the desire for prosthetists who listen to and understand women’s needs.

**Conclusions:**

Women Veterans with LEA articulated themes reminiscent of those previously reported by male Veterans with LEA, such as the importance of prostheses and the central role of the provider-patient relationship. However, they also articulated unique needs that could translate into specific strategies to improve prosthetic care, such as integrating formal opportunities for social support and peer interaction for women Veterans with LEA, advocating for administrative changes and research efforts to expand available prosthetic component options, and ensuring that clinical interactions are gender-sensitive and free of bias.

**Supplementary Information:**

The online version contains supplementary material available at 10.1007/s11606-022-07572-8.

The United States (US) Department of Veterans Affairs (VA) is a world leader in amputation care and serves a heterogenous group of Veterans. Women Veterans with amputation have grown in number and proportion, increasing by 28% from 2,049 to 2,622 Veterans (or from 2.3 to 2.7% of all Veterans with amputation) from 2015 to 2019.^1^ Despite their small number, women with amputation have been recognized as a critical subpopulation with unique needs. In 2017, VA designated prostheses (devices that support or replace a body part or function for purposes of increased mobility and function) for women Veterans a national research priority, and in 2020, the Government Accountability Office produced a report on VA’s efforts to provide and study prostheses for women Veterans.^1^ Despite these national priorities, study samples in VA research on prostheses have typically included less than 5% women, usually with sample sizes under ten.^2–8^ While this accurately reflects their proportion in the population, the data have been insufficient to adequately characterize women or explore potential gender differences. Thus, prosthetic advances for Veterans with amputation have been primarily designed and optimized to meet the needs of men.

In the general population, women with lower extremity amputation (LEA) appear to have worse functional outcomes than their male counterparts. Women with LEA report poorer physical functioning and worse health-related quality of life compared to men with LEA.^4,9–12^ These outcomes are intertwined with prosthetic practice. Studies of women with amputation found that they were less likely to use a prosthetic limb than men and reported greater dissatisfaction with their prostheses.^13,14^ In one of the only studies to date comparing men and women Veterans with amputation, women reported higher rates of receipt of prostheses but also greater rates of rejection and lower rates of replacement of prostheses than men.^15^ However, the sample was limited to 283 Veterans who served in Iraq and Afghanistan, of whom only nine were women, limiting the generalizability of these findings.

To increase the evidence base related to women Veterans with LEA, we conducted qualitative interviews to understand their experiences with prosthetic care and their prostheses. Given the lack of research on this group, we aimed to approach women’s experiences in an exploratory fashion, with the broader goals of identifying directions for future research and strategies to improve clinical practice.

## METHODS

### Sampling and Recruitment

We identified women who had an acquired major LEA (defined as amputation at the ankle or more proximal level), due to traumatic or nontraumatic etiology, using procedure or diagnosis codes in the national VA electronic health records (EHR). We mailed prospective participants a letter describing the purpose of the study, how to participate, and how they could opt out of further contact. We made follow-up calls and sent one additional letter to explain the study and assess interest. Eligibility, which included having been prescribed a prosthesis at least 12 months prior, was confirmed for those who expressed interest.

Eligible women who agreed to participate provided verbal informed consent. Procedures were approved by VA Puget Sound Health Care System Institutional Review Board.

### Data Collection

In-depth, semi-structured qualitative telephone interviews were conducted by three study authors (KL, RT, AL) between October 2019 and March 2020. Interviewers were women, had a master’s or doctoral degree, and were trained in qualitative data collection methods. Interviewers used a written guide designed to start with broad, open-ended questions, followed by more specific questions and probes to elicit detailed descriptions of women’s experiences with prostheses, clinicians, and systems of care. Participants were asked to describe their experiences with the prescription and fitting of their prostheses; their clinical encounters and training experiences related to their prosthetic care; and their use of, and satisfaction with, their prosthetic limb(s) (see Supplemental Table 1 for Interview Guide). Interviews were digitally recorded, transcribed verbatim, and reviewed by the qualitative team lead (JY) for quality assurance. The qualitative lead provided interviewer feedback and assisted with refining the interview guide to ensure high-quality data collection. Data were collected until thematic saturation, at which point two more interviews were conducted to ensure no new themes or concepts emerged.^16^

Additionally, to describe the population, we used data from the EHR (e.g., age, race, marital status, service connection) and from self-report (e.g., amputation level and cause, year of first amputation, prosthesis use). Veterans’ current residences were grouped into major geographic regions.^17^

### Analysis

Data were analyzed using inductive content analysis, an approach in which themes are derived through open coding, code grouping, categorization, and identifying patterns of meaning across the data.^18,19^ The study team reviewed and discussed all transcripts to create a code list based on meaning units identified in the data. The qualitative team lead (JY) applied the coding framework to the data. The analytic team (KL, JY, RT, AL) met frequently to review coding application, refine code definitions, and add new codes. Disagreement was resolved through discussion. Data were sorted into categories based on similarities and differences within and across interviews; themes were identified based on patterns. All analyses were conducted using Atlas.ti software.^20^

Interview data were iteratively revisited by the analytic team to further refine themes, ensure that findings were grounded in the data, and validate results.^21^ Preliminary findings were reviewed by the entire study team, which included clinicians and researchers from multiple disciplines with expertise in amputation and women’s health, at multiple time points, to finalize themes, confirm the validity and credibility of findings, and identify representative quotes.

## RESULTS

### Participants

**Fig. 1:**
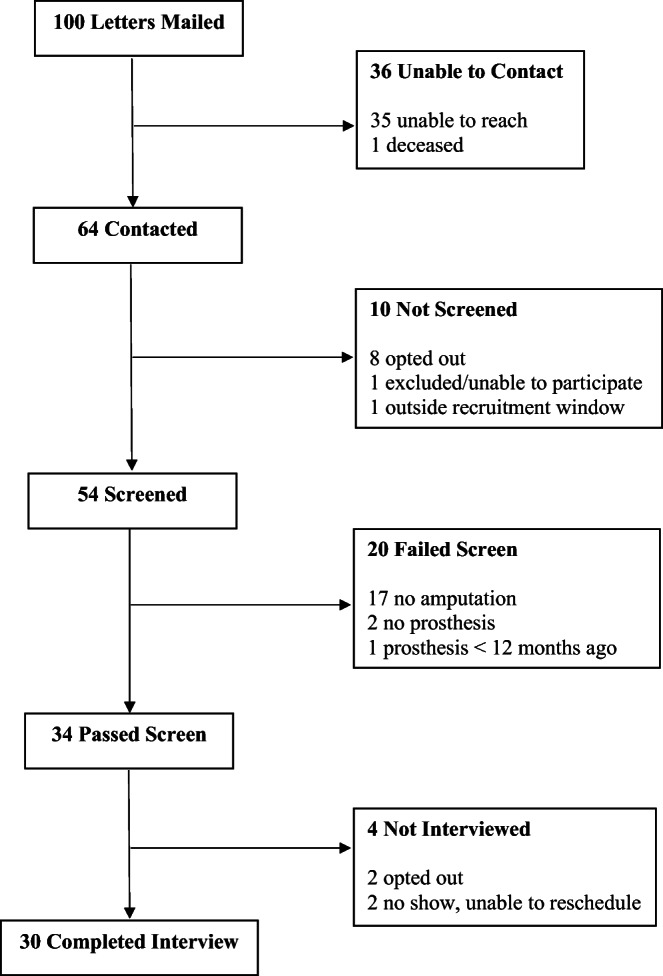
Study flow chart

We mailed letters to 100 Veterans, screened 54, and completed 30 interviews (see Fig. 1). Participants were diverse on demographic and amputation-related characteristics (Table 1). Most reported using their prosthesis at least some of the time (66%), with 33% not currently using it. Common reasons for ceasing to use a prosthesis included pain, complications related to prosthetic use (e.g., skin breakdown, stump infection, musculoskeletal problems), fear of or history of falling, surgical revision, clinical advice, lack of success using the prosthesis in desired situations, and acceptance of a wheelchair as an easier or better alternative.

**Table 1:** Characteristics of Interviewed Women Veterans with Major Lower Extremity Amputation (*N* = 30)

Variable	*n* (%)
Age, years	
< 40	2 (7%)
40–49	2 (7%)
50–59	6 (20%)
60–69	12 (40%)
70–79	5 (17%)
80+	3 (10%)
Race	
Black	3 (10%)
Other	3 (10%)
White	24 (80%)
Marital status	
Divorced	14 (47%)
Married	5 (17%)
Never married	8 (27%)
Widowed	3 (10%)
Current residence by geographic region	
Midwest	5 (17%)
Northeast	1 (3%)
Southeast	6 (20%)
Southwest	6 (20%)
West	12 (40%)
Service connection^a^	
Not service connected	15 (50%)
0–40%	0 (0%)
50–90%	9 (30%)
100%	6 (20%)
Amputation level	
Transtibial	14 (47%)
Transfemoral	15 (50%)
Bilateral^b^	1 (3%)
Amputation etiology	
Trauma	11 (37%)
Peripheral vascular insufficiency/diabetes	9 (30%)
Infection	7 (23%)
Other^c^	3 (10%)
Time since amputation, years	
1–2	4 (13%)
3–4	9 (30%)
5–9	6 (20%)
10–19	4 (13%)
20+	7 (23%)
Currently uses prosthesis	
No	10 (33%)
Yes	20 (66%)

^a^Service connection is determined in 10% increments, between 0 and 100

^b^Bilateral amputations included a transfemoral and a transtibial amputation

^c^Includes unknown etiology, medical error, and failed knee replacement

Four key themes and three subthemes emerged related to women’s experiences around prostheses and prosthetic systems of care. Exemplar quotes are provided; additional supporting quotations are included in Supplemental Table 2.

#### Theme 1

**“I don’t know anyone like me”: Women with LEA reported feeling “invisible” and lacking connection**

Participants commonly described themselves as “invisible” and reported never seeing other women with amputation or prosthetics. As one woman (P18) explained, “I see a lot of men. And I see very few women. I can’t remember the last time I saw a female either in the VA system or outside the VA system with a prosthetic device. …. It’s kind of like a true minority.”

Almost all women described a desire for connection with “others like me who will understand me and my experiences,” yet very few had ever met or known anyone like them and were unaware how to find other women with LEA. Women described a range of anticipated psychosocial, informational, and practical advantages of peer contact including access to role models, reduced isolation, increased self-confidence and positive self-image, resources and information sharing (e.g., where to find shoes, prosthetic options, how to find a good prosthetist), and education and support around new challenges (e.g., getting around with a prosthesis, body image, intimacy and relationships, and the effects of menopause and aging on prosthetic fit).

Would be helpful…knowing other females who are amputees or having groups or something like that…somebody to come along side and teach [women]… just educate them as far as, yes, you can wear these kind of shoes… Education on how to get the right leg, the right doctor. Learning self-confidence again. …Learning how to deal with your body with missing parts to it. It’s rebuilding that confidence that we’re still a whole person in spite of missing a limb. (P25)

Participants explained that although programs to foster contact among women with LEA were wanted, such as virtual or in-person gender-specific peer support and educational groups, existing services often failed to meet women’s needs. Women who had attended amputee groups at VA medical centers often described themselves as “out of place” and “uncomfortable” as the only woman in attendance, and these male-dominated amputee groups failed to provide the support, targeted information, and guidance women desired. Additionally, some educational materials shared at groups were designed for a male audience and failed to represent women, further contributing to women’s feelings of invisibility. For example, P2 described male-oriented sexuality resources provided at an educational group for Veterans with amputations: “They had pictures and every one of them was a man amputee. … That they wouldn’t even think about that, there’s women soldiers. …made me mad…I’m old, but I’m not dead.” Some women described current services as “unfair,” “lacking,” or “unequal,” and called upon VA to ensure its amputee programs met the needs of women Veterans with LEA.

#### Theme 2

**“They are made for men”: Prosthetic limbs and components often did not meet women’s needs**

Women explained that having “the right leg made a world of difference” and “opened up the world” through allowing them to participate in valued activities, increasing quality of life, and affecting the way others viewed them. However, many reported being prescribed prostheses that were “designed for men” and neither fit their bodies nor met their physical needs. Women often described difficulties in getting “a leg that works for me” and design and fit issues, such as heavy prosthetic limb weight and problematic socket size and fit. Ill-fitting lower limb prostheses often resulted in pain, skin breakdown, blistering, and chronic problems with the hip, back, and/or contralateral limb and negatively impacted women’s balance confidence. As P24 explained, “They never could get the fit right on my stump…in the bucket you know. Just never really fit me, kept on rubbing and causing wounds and problems…he [prosthetist] said that they are made more to fit men.”

Women often expressed frustration with their lack of prosthetic options compared with their male counterparts. Some described requesting prosthetic legs or feet that they had seen in catalogues or on men with LEA only to be told that those prostheses were unavailable or did not come in their size. When women expressed dissatisfaction with prescribed prostheses or asked for additional choices, some were told “there is nothing we can do” and that the system lacked options that fit female bodies. This explanation was often accompanied by a “take it or leave it” attitude, suggesting women were expected to accept the provided prosthesis despite its limitations or to function without one.

So, I told him, I said, this leg is too heavy for me. He said ‘Well, that’s your only choice. You don’t get a leg. Take it or leave it’ ...If you say that it [prosthesis] doesn’t work, it’s like, ‘Well nobody else has a problem, just be grateful for what you get’. (P15)

#### Subtheme

**“Just make something that works for us…and give us the options”: Recommendations for prosthetic design**

Many participants offered suggestions for improving prosthetic design to better meet women’s needs. Women wanted prosthetic limbs that were lightweight, less bulky, and available in “women’s sizes” and sockets that were optimized for women’s bodies. Many described a need for prostheses that “don’t tear up your clothes” with narrower, more form-fitting, softer sockets to allow women to wear a wider range of clothing options. Women also desired modification to better meet changes related to hormonal shifts, menopause, and aging. These included sockets that could be adjusted by patients in response to weight fluctuation and swelling and absorbent socket materials or socket inserts to address sweating. A few women also reported needing prostheses that better met their toileting needs. P12 explained, “We have to sit more than men do, so when you have to use the restroom that leg falls off.”

Women commonly described a need for prosthetic foot options that would accommodate a variety of types of shoes. Prescribed prosthetic feet often limited women’s choices to “tennis shoes or other tie-up shoes” despite some women’s desire to “be able to wear heels, dressy shoes, sandals and other girly-shoes like that.” Women were interested in prosthetic feet that would accommodate shoes with varying heel heights and split toes to expand shoe choices and improve confidence across a broader range of settings and roles.

Participants also underscored the need for transparent, accessible information on and greater consistency in availability of different prosthetic options across facilities. Women described great variability in the type and range of prostheses provided at VA facilities and a lack of publicly available information on approved prosthetic socket, knee, and foot options. Lack of information limited women’s agency and left them “at the whim of what someone else wants you to get…wants you to have.” Many women “did not know where to start looking” for options and believed that prosthetists had a responsibility to provide comprehensive information on “as many options as I can get…whatever is available to me as a Veteran.”

#### Theme 3

**“You need to know who the woman is and what her goals are”: The need for individualized assessment in prescribing**

Participants described the need for a patient-centered approach in which clinicians “get to know who you are…the whole woman…all about you” and “ask you what you want and need in a leg” to guide prescribing. Knowing the “whole woman” was viewed as essential to “getting the leg you want [that] you can use the way you want to use it,” and included assessment of prior and desired level of functional ability, roles and responsibilities, interests, medical history, mental health, and social support. However, few women reported being asked about their history, needs, or goals prior to prosthetic prescription.

Participants commonly emphasized that “not all women are the same” and described ways in which they differed from a “typical woman” as a backdrop for explaining what they wanted in a prosthesis. Some described encounters in which clinicians expressed gender-based stereotypes that affected prosthetic selection and prescription. For example, some reported asking for prosthetics that would allow them to engage in outdoor activities only to have those goals questioned or dismissed by clinicians.

Like the last time when I went to the Amputee Clinic, I said, ‘I like to go fishing and I would like to go canoeing a little bit and stuff, but I can’t get this prosthesis wet, is there a type of prosthesis I can get wet? Or is there a cover or something that can be put over it?’, and he just looked at me and he said, ‘so **you** want to go fishing?’ (P1)

Participants also described experiences in which clinicians explicitly or subtly emphasized prosthetic appearance over function in selection and prescribing.

Well initially, I had the type of prosthesis that you had to hold on with a belt…It was quite limiting, you had to do all of the old layers and layers of stump socks…It was just so cumbersome and so difficult and get around. When I would talk to the prosthetist about it, their attitude was, ‘women don’t really care about that, you just care that it looks good.’ (P30)

#### Theme 4

**The prosthetist is key to “making your leg fit well” and “work for you”**

Women reported that prosthetists played a central role in getting their prosthesis to fit well, adapting prostheses to work for women’s bodies, identifying and advocating for prosthetic options, and “figuring out how to make my leg work for me.” Women reported a range of experiences with prosthetists, from very positive to very poor, and great variation in prosthetist quality, caring, and skill. Having the right person to work with was particularly important to women who described themselves as difficult to fit and some suggested that fitting women may take more time, skill, and effort given gender differences in anatomy and a lack of prosthetic components designed for women.

As a woman…I am one of those difficult to fit people…because I’m short I’m not compatible with everything, because you require a certain length in order to be able to use certain types of feet, so I’d never found anything to really work for me …so I have to have someone who is really willing to go to bat for me to find something that will work. (P29)

#### Subtheme

**Women want prosthetists who “listen,” “take me seriously,” and “work with me”**

Participants described three central qualities that defined a “good prosthetist” and were associated with prosthetic and clinical satisfaction. First, women wanted to be listened to when they voiced questions, concerns, or needs. Second, participants wanted concerns to be taken seriously, as demonstrated when prosthetists made a concerted effort to find a solution, adaptation, or option that addressed their concern. Finally, women wanted prosthetists to work with them in obtaining the best possible prosthetic fit and function. Effective prosthetists gathered information on fit and function (e.g., “watched me walk in it,” “had me try on a couple options”), elicited feedback, and made adjustments until the woman was satisfied or “it was as good as it could get.”

#### Subtheme

**We want “someone who understands women”**

Participants shared that they wanted better access to female prosthetists who could relate to their needs, and “would know how to work with women amputees.” Women described a lack of female clinicians in prosthetic departments and amputee clinics, and some believed that increasing gender representation and equity in the clinical setting would change the “face and culture” of prosthetics services, resulting in more gender-sensitive care.

In my group of people through the Prosthetics Department in the VA, there’s only one woman in the group of people. Everyone else is a man…they don’t deal with PMS and swelling from bloating. So they have no idea why there are days I can’t put my leg on…They have no idea how to deal with that. And even if they’re an amputee, they still don’t know, that’s not something they deal with….The men have no idea. So I think having more women included would be beneficial to women amputees. (P13)

Women also recommended education and training for prosthetists to increase their knowledge and skill in fitting and adapting prostheses for women and cultural competency in working with women with LEA. A few participants suggested prosthetists should be certified to work with women, particularly those with above-the-knee amputations, so women could trust that the prosthetist had the comfort, sensitivity, and skill for successful fitting.

It’s really hard to find a good prosthetist. And a lot of the VA people are so used to dealing with men, that I don’t think they put the consideration in for women…. if there was just a way to pre-qualify them to see if they actually helped women with an above the knee. …It’s so up-close and personal, you have to have somebody that’s really comfortable and good working with women. (P6)

## DISCUSSION

To our knowledge, this is the largest qualitative study conducted to date with women Veterans with LEA. Some findings echoed existing knowledge on primarily male Veterans with LEA, such as the importance of prostheses for quality of life^22^ and functional mobility,^23^ the need for patient-centered and individualized assessment,^24,25^ and the central role of the provider-patient relationship for optimal success.^26^ Other themes reflected the unique challenges facing women Veterans with LEA, such as feelings of invisibility, desire for connection with other women with amputation, lack of prosthetic options that fit women’s bodies and needs, and bias and discrimination in clinical encounters.

Prominent in the data was the sense of isolation reported by women Veterans with LEA. Both perceived isolation (e.g., loneliness, perceived lack of social support) and social disconnectedness (e.g., small social network, infrequent participation in social activities) have been independently linked to poorer physical health, and the former to poorer mental health.^27,28^ Our findings highlight the need to further examine isolation, loneliness, and social support—and their potential impact on health outcomes—in women Veterans with LEA. For example, future quantitative research could evaluate differences in social support between men and women Veterans with LEA and assess whether potential differences in support drive other psychosocial and health outcomes.

Findings also suggest that existing social support resources are inadequate for women. While many VAs offer amputee support groups, most are limited to a single geographic area and are thus predominantly or exclusively male. Developing and expanding telehealth options for peer connection, such as virtual support groups, could expand access to services for geographically dispersed women with LEA. In addition, the VA Amputation System of Care has a Peer Visitation Program for Veterans. The program could be targeted to train more women Veteran peer visitors to allow for gender-specific connection.

Women expressed frustration that available prosthetic options failed to meet their needs. For example, problems with fit and prosthesis weight were frequently discussed, which in turn led to dissatisfaction, wounds and infections, problems with mobility, and reduced use of the prosthesis. Women viewed poor fit to be a result of prostheses being designed for men, without taking their body sizes and transient changes (such as pregnancy, menopause, and/or monthly weight fluctuation) into account. Moreover, prosthetic components often did not allow for flexible clothing or shoe options, which for some women was important for their social roles, responsibilities, and identity. Design innovations are needed to address these issues. This is a nascent area of research; recent work has begun to examine novel solutions to expand the range of available footwear options for women.^29^ Providers and prosthetists working with women Veterans may play a role by advocating for more tailored options, which may require those more centrally involved with prosthetic design, engineering, and development to take action.

Women recounted experiences of explicit and implicit bias in clinical encounters in which providers made gender-based assumptions about their preferences, needs, and interests and failed to solicit or dismissed their input. These experiences demonstrate the need for making care more gender-sensitive. The VA/DoD Clinical Practice Guidelines for Rehabilitation of Individuals with Lower-Limb Amputation acknowledge the importance of gender and specifically include a recommendation to consider “birth sex and self-identified gender identity in developing individualized treatment plans.”^24^ While this is a critical first step, our findings suggest gaps in successful, consistent implementation of these guidelines and opportunities for improvement. For example, provider trainings and educational interventions may be needed. A recent systematic review on gender-sensitivity educational interventions for healthcare providers found a promising trend toward improvement in gender-related knowledge, attitudes, or practice after intervention, although overall there was insufficient evidence to determine effectiveness.^30^ The VA has been a leader in producing trainings to promote sensitivity in working with a variety of minority groups (e.g., LGBTQ Veterans); tailored trainings on women Veterans with amputation may be called for across rehabilitation services. Implementing consultation services for providers or directly connecting patients with women’s health experts may be additional approaches worthy of consideration.

The study has limitations. As we recruited women Veterans enrolled in VA, findings may not generalize to civilians or Veterans outside of the VA. Although all participants were enrolled in VA, not all prosthetic care occurred in VA. We did not consistently ascertain detailed context for each clinical encounter, such as system of care (VA vs. community) and care location (rural vs. urban). Care environment may impact prosthetic experience and should be explored in future research. Additionally, some clinical experiences shared by participants occurred in the distant past, so it may be difficult to ascertain their relevance to the current context of care. While this study focused on women Veterans with LEA, the experiences of those with upper extremity amputation should also be evaluated.

In conclusion, this qualitative study with women Veterans with LEA revealed a number of gender-specific themes related to prostheses. Critical areas requiring attention include the need to bolster social support and peer interaction, advocate for and expand access to prosthetic options, and ensure clinical interactions are gender-sensitive and free of bias. The VA and other systems of care have an opportunity to expand and tailor services for this population and invest in provider resources and education. Doing so will help ensure that their voices are heard and prioritized.

## Supplementary Information

ESM 1 (DOCX 14 kb)

ESM 2 (DOCX 23 kb)
