# Supplementary material for: Voices of Women Veterans with Lower Limb Prostheses: a Qualitative Study
Source: J Gen Intern Med. 2022 Sep 1;37(Suppl 3):799–805. doi: 10.1007/s11606-022-07572-8 (PMC9481864; doi:10.1007/s11606-022-07572-8)
Supplement: Supplementary file 1 — (DOCX 14 kb) [file 11606_2022_7572_MOESM1_ESM.docx]

**Supplemental Table 1. Interview Guide.**

Interview Questions

**Can you give me a brief history of what caused your amputation and your history with your prosthesis or prostheses?**

**Tell me how things have been for you since your amputation.**

Tell me about getting around.

Tell me about what you can and can’t do since your amputation.

**Tell me about something you have been able to do (that seemed hard at first or took some time to be able to get back to).**

**Tell me about something you’d like or need to do but haven’t been able to since your amputation.**

**Tell me about [*or tell me more about*] your experience with your prosthesis.**

How does your prosthesis help/not help you get around?

How does your prosthesis help/not help you do the things you want to do?

How has your use of your prosthesis changed since you first got it?

**Tell me about getting your prosthesis.**

Tell me about what it was like first getting it.

Tell me about working with your prosthetist.

Tell me about getting a prosthesis made for you.

**Tell me about learning to use your prosthesis.**

**Have there been times when you stopped or considered stopping using your prosthesis?**

If yes: Tell me about one of those times.

If no: Are there times when you find yourself not using your prosthesis or not wearing it? Tell me about one of those times.

**What could be done to improve the experience for women with an amputation?**

How could the VA better help women with an amputation?
What feedback do you have for prosthetists?
What feedback do you have for your doctors?

What feedback do you have for prosthetic designers?

**Is there anything else that is important for us to know about getting back to doing the things you want and need to do after an amputation?**

Standardized Probes

*Probes should use words or phrases presented by the participant using one of the following formats:*

- *What do you mean by ____________ ?*
- *Tell me more about ____________ ?*
- *Give me an example of ____________ ?*
- *Tell me about a time [last time] ____________ ?*
- *Walk me through _______.*
- *Who/when/where ________?*
- *How did x compare with y?*
- *What got in the way of _________?*
- *What helped with _________?*
- *What has been positive about ___________?*
- *What has been challenging about ___________?*
- *What could be/have been improved about ________?*
