# Supplementary material for: Voices of Women Veterans with Lower Limb Prostheses: a Qualitative Study
Source: J Gen Intern Med. 2022 Sep 1;37(Suppl 3):799–805. doi: 10.1007/s11606-022-07572-8 (PMC9481864; doi:10.1007/s11606-022-07572-8)
Supplement: Supplementary file 2 — (DOCX 23 kb) [file 11606_2022_7572_MOESM2_ESM.docx]

**Supplemental Table 2. Additional Exemplar Quotes By Theme**

| **Theme 1. *“I don’t know anyone like me”*: Women with LEA reported feeling “*invisible”* and lacking connection** | |
| --- | --- |
| *I’ve never known a female amputee…I don’t know anyone like me in that way, who is an amputee…never seen one at VA or talked to one that I know of…wouldn’t know how to meet someone like me…but wish I could.* | P9 |
| *I never had any female role models. If there were more female amputation role models for female amputees, that would be extremely helpful. To help learn and discuss what do you do …you know, being intimate with another, with a man or a woman. Being intimate with another person, given your loss. How do you deal with your self-image?* | P21 |
| *Meeting somebody in person…others like me who will understand me and my experiences… to share some peer to peer advice on how we’re navigating life as an amputee and what’s been helpful for us, and some challenges that one amputee may have, that another amputee may have figured out how to overcome.* | P19 |
| *There were two different types of classes or groups, and I didn’t have anything in common with any of them [men]…none of them were women. I stuck out you know…it was uncomfortable to be the only one and it wasn’t really helpful to me at all.* | P14 |
| **Theme 2. *“They are made for men”*: Prosthetic limbs and components often did not meet women’s needs** | |
| *Having the right leg made a world of difference. It just kind of opened more things to me…opened up the world to me more. And I could walk faster, get out better… do the things I wanted to do… And it just opened up a whole different level of activity for me that I wasn’t able to do before.* | P1 |
| *It put me on an even playing field. Just to be able to pop my leg on in the morning and take off, and not really think about it until bedtime, when it was time to take it back off. In fact a lot of people that didn’t know me, that met me, they’d go, ‘oh my gosh, you’re an amputee?’. They had no idea, they didn’t see an amputee, just a normal woman.* | P8 |
| *They told me in [city] that the socket that I got came from [different city] and was more designed for men, because they don’t have quite the pubic curve that we do, females do. So that was always riding on the pubic curve bone. Which I find, there’s a lot of older men that go to this shop, I don’t know if there’s any other female amputees that have gone to it, so I think that they’re just in that rut where they’re used to dealing with older men.* | P6 |
| *It just didn’t fit right and it was way too heavy…had to swing my leg out to walk and after doing that for long enough my other leg started having problems…and my hip and back got messed up and then my back cause I was walking funny…caused me lots of other problems.* | P4 |
| *It’s like this…asked a guy I know what kind of leg he was using because he could do all sorts of things with it…but come to find out they don’t make it in my size. … Seems like that’s what I always hear, “It doesn’t come in your size”.* | P15 |
| *He said it’s the one style that they have, and that this is what they go with, and that it’s their normal practice and procedure. I said, ‘you know what? I get tired of you guys telling me that this is it, this is it, this is it, there’s nothing we can do, we deal with more men than we do women. There’s a lot more women veterans out there than there used to be’.* | P11 |
| ***Subtheme. “Just make something that works for us…and give us the options”:* Recommendations for prosthetic design** | |
| *Maybe they could make them lighter and in different sizes…women’s sizes you know? I’m not a big person and I need one that I can actually move around in and actually comes in my size for shorter people, you know? … Not everyone is six feet tall…Just make something that works for us, and give us the options…let us have the options that work for us.* | P1 |
| *I have this really thick bucket. It’s really thick and really hard. I see people with smooth and soft. Why can’t I [have a thinner one]? I can’t wear any of my clothes if I have the prosthesis on, because the bucket is so big.* | P3 |
| *The problem with the fit is that my weight goes up and down throughout the month …get swelled up and then down so then the leg wouldn’t fit right…they don’t make them so you can adjust them the way you need to. … it keeps me from being able to use my leg the way I’d like to.* | P7 |
| *I’d found out that there were feet that had a split toe so I could actually wear sandals….so I wore dresses all summer long and that foot also had an adjustable heel, so I didn’t have to just wear one pair of shoes. I could switch between sandals and if they weren’t the perfect height then I could adjust the heel. That’s a huge thing. To the women that I talk to. Men basically wear one pair of shoes. … If I would have known all that stuff in the beginning, I think I would have advanced and felt better mentally in the beginning knowing that I could change my shoes*. | P30 |
| *The prosthetic company that I was working with at the time, I think they were very … they were the only one that had been in this area for years. they were pretty stuck in their ways and didn’t tell you all the options that you had. It was like, here’s the foot and here’s the leg…they wouldn’t tell you about options…it’s like you’re at the whim of what someone else wants you to get, what someone else wants you to have.* | P27 |
| *If there are other prosthetic devices that are on the market then the VA should have them available to you instead of just one single type of prosthetic device. Need to give you all the information ….I want as many options as I can get…whatever is available to me as a Veteran.* | P18 |
| **Theme 3: *“You need to know who the woman is and what her goals are”*: The need for individualized assessment in prescribing** | |
| *They need to ask the person what they want and need in a leg…It should be specifically geared for what your needs are and how active you are. And what you need a leg to do. If you’re involved in sports or you walk a lot, or you carry a lot of things or you have specific needs that you need to be able to stand for, and they don’t have that. That’s missing from the equation.* | P20 |
| *My feedback would be to take a little time to actually look at all of the patient’s information, and have a bigger picture, get to know who you are….the whole woman, everything you know, all about you. Not just, ‘this is the meeting we’re having today’. So have the information with you and look at, what does this person want?* | P13 |
| *And to me, if they know that you have an anxiety disorder, if you have other medical problems, they need to know that before they work with you to get up and find you the right kind of leg to get you. They need to know if you have sciatic nerve problems. They need to know more of your medical history and medical problems before they just throw at leg at you and say, ‘here’.* | P30 |
| *Well not all women are the same you know, they shouldn’t treat us that way. Like me, I’m not a typical woman…don’t care about how the thing looks, just want it to be able to keep up with me because I’m really active.* | P19 |
| *I think one of the things the VA could do would be to focus on functionality, and not just on appearance. And I think they have made strides in doing that over the years, but there’s still a little bit of a, ‘well, you’re a woman, it really doesn’t, do you really need that?’, type of thing…if I go in and say, ‘I would kind of like to try this because I want to climb on a ladder’, they’d be like, ‘well what are you going to be on a ladder for? You don’t need to be on a ladder.’…So I think there’s still a little bit of a stigma. And I think just the attitude that if it fits, not that it fits well, but if it fits.* | P11 |
| *I was at the VA in [city] one time, and they were doing this study where they connected sensors to your shoes, and they were having you walk back and forth so they could see what your gait was and all of that. The technician was going to do it with me, and she had me all hooked up and everything, and the prosthetist came out and he said, ‘what are you doing?’, and they said, ‘we’re going to have her do that’, and he said, ‘no, you don’t need her to do that, it doesn’t make any difference, as long as her leg looks good, that’s all that matters’* | P5 |
| **Theme 4. The prosthetist is key to “*making your leg fit well” and “work for you”*** | |
| *Who you work with, that is the most important thing to getting a leg that works and let’s you do what you want to do. [Name], he has been the most important thing to get a leg that I can use and figuring out how to make my leg work for me.* | P26 |
| *That first one [prosthetist] he was just doing a job, you could tell, told me that was as good as it was going to get…[name], I started seeing him at a different VA, he said ‘we’re going to work this out’ and he did…he went above and beyond to make sure that it [prosthetic] didn’t hurt me ..made sure it fit me and I could get around so much better.* | P27 |
| *You have to go through them [prosthetists] to find one who will listen to you and actually help you out…before I found [name] I had some who just didn’t care, didn’t want to make any effort to make it work for me…now because of [name] I’m not in pain and can do what I want to do.* | P7 |
| ***Subtheme. Women want prosthetists who “listen”, “take me seriously”, and “work with me”*** | |
| *So, having him listen to what I have to say about what’s affecting me. He’s really been proactive with the [prosthetic] company about what is going on with my new knee…. If anything goes wrong with the knee, [company] calls him and they talk it over and things are worked out. He’s really a good guy and he takes me seriously, what I think, what I need.* | P26 |
| *He was the only one that figured out a way to fix that leg where I didn’t slide off the toilet. He listened to what I had to say and said that yeah, it does make sense, women do have to sit down to use the restroom more than men do. So he actually adapted the leg to a female, because those things are slippery. So he actually listened and took what I said seriously.* | P12 |
| *It wasn’t a point of just you know, “Here’s your leg”. It was going out and walking and when I would come from my appointments, he’d come out the reception area and get me and walk me back. And he watched me walk in it and he could visualize whatever adjustments had to be made from the way I was walking. It was just a lot more… he had me try on a couple options, different options….he demonstrated a lot more knowledge and more care because they were really watching what they thought you needed. Not what they thought just would do the job.* | P25 |
| *They’re not just going to let you walk out, they’re going to make sure you’re satisfied with the product and everything, that you’re not having pain, if anything goes wrong, they’ll say, ‘go home, walk in it for a week and if something is not sitting right, you come back”. They want to know how it is working for you. And those are the kind of people I prefer to work with, because they do actually care about getting you back up on your feet and doing the things that you want to do.* | P17 |
| *With the prosthetic, they worked with me and they made it to where it wasn’t so bulky, and I was able to actually get up on my bike and ride and do the things I love to do. They did a lot of adjustments for me, they really really worked with me a lot to make everything so that it would be enjoyable for me to have a prosthetic leg.* | P20 |
| ***Subtheme. We want “someone who understands women”*** | |
| *I just feel like that maybe they need to have a female prosthetician who deals with females who can relate to what your needs are. Because me and this guy who’s making the legs over there, we don’t have anything in common. The only thing in common is that he works at VA and I go to VA.* | P18 |
| *I would just like to see the VA do more for women amputees than they do. I know there are more men in the military and more men retirees, but there’s a lot of women out there that could use help too. It would be nice if we had our own clinic, our own doctors to talk to. Female doctors. Right now, all of my doctors are male. They don’t understand crap that I try to tell them. When I have a problem, it’s like talking to a brick wall, because they don’t really understand. …It’s like I said, they need more female doctors.* | P10 |
| *If we had more women doctors [in prosthetics], it would basically change the face and culture of VA…it would make a big difference…not just for me but to keep women’s needs more at the forefront in general…so that everyone does better by women amputees, that we get what we need.* | P21 |
| *They need to get the doctors together and do more training [about working with women amputees]… I know there’s a lot of culture programs where people come in and they’re experts, and they talk to people to deal with women appropriately …this is something that they need to be going through.* | P22 |
